# Water scooping: tool use by a wild bonobo (*Pan paniscus*) at LuiKotale, a case report

**DOI:** 10.1007/s10329-024-01121-z

**Published:** 2024-03-15

**Authors:** Sonya Pashchevskaya, Barbara Fruth, Gottfried Hohmann

**Affiliations:** 1 Max Planck Institute of Animal Behavior, Constance, DE Germany; 2 Centre for Research and Conservation, Royal Zoological Society of Antwerp, Antwerp, Belgium

**Keywords:** Tool use, Bonobo, Water drinking, *Pan paniscus*

## Abstract

Tool use diversity is often considered to differentiate our two closest living relatives: the chimpanzee (*Pan troglodytes*) and the bonobo (*P. paniscus*). Chimpanzees appear to have the largest repertoire of tools amongst nonhuman primates, and in this species, many forms of tool use enhance food and water acquisition. In captivity, bonobos seem as adept as chimpanzees in tool use complexity, including in the foraging context. However, in the wild, bonobos have only been observed engaging in habitual tool use in the contexts of comfort, play, self-directed behaviour and communication, whilst no tool-assisted food acquisition has been reported. Whereas captive bonobos use tools for drinking, so far, the only report from the wild populations comes down to four observations of moss sponges used at Lomako. Here, we present the first report of tool use in the form of water scooping by a wild bonobo at LuiKotale. An adult female was observed and videotaped whilst using an emptied *Cola chlamydantha* pod to scoop and drink water from a stream. We discuss the conditions for such observations and the importance of looking out for rare behaviours and attempt to put the observation into the context of the opportunity versus necessity hypotheses. By adding novel information on tool use, our report contributes to the ongoing efforts to differentiate population-specific traits in the behavioural ecology of the bonobo.

**Supplementary Information:**

The online version contains supplementary material available at 10.1007/s10329-024-01121-z.

## Introduction

The frequency, complexity and diversity of tool use has often been considered as one of the domains that differentiates our closest living relatives, the chimpanzee, *P. troglodytes*, and the bonobo, *P. paniscus* (Gruber et al. 2010; Koops et al. 2015). Among nonhuman primates, chimpanzees appear to have the largest repertoire of tools (Whiten et al. 2001). While there is considerable variation across populations, all populations that have been studied engage in tool use to various extents (Whiten et al. 2001) and use some types of tools habitually (McGrew 2004); i.e., some of the individuals routinely exhibit the behaviour (Whiten et al. 2001). Definitions of tool use in animal literature have varied greatly over the years, and there have been many attempts of refinement (Bentley-Condit and Smith 2010). We will use the definition offered by Shumaker et al. (2011): “The external employment of an unattached or *manipulable attached* environmental object to alter more efficiently the form, position, or condition of another object, another organism, or the user itself, when the user holds, *and directly manipulates* the tool during *or prior* to use and is responsible for the proper and effective orientation of the tool” (emphases by Shumaker et al. 2011). In chimpanzees, many forms of tool use enhance access to food (Whiten et al. 2001; Sanz and Morgan 2010), although its effect on fitness remains to be assessed (Biro et al. 2013). Compared with this, evidence for tool use by wild bonobos is still scarce. The two forms that have been reported from all field sites include construction of tree nests and the use of drag branches (Hohmann and Fruth 2003). Preliminary data compiled from reports of different populations suggest that bonobos’ tool use repertoire falls within that of chimpanzees and that, for the two species combined, the number of different types of tool use is similar across the different populations (Furuichi et al. 2015). Moreover, in captive settings, bonobos appear to be as adept as chimpanzees with respect to tool use diversity and complexity (Jordan 1982; Gruber et al. 2010), the latter referring to the number of behavioural components, actions’ sequencing and objects’ organisation (Sanz and Morgan 2010). However, what differentiates bonobos and chimpanzees in the wild is not only the frequency of tool use but its context: in chimpanzees, the majority of reported tool use occurs in the context of feeding and foraging, whereas in bonobos, it is associated with play, comfort, self-directed behaviour and communication (Ingmanson 1996; Furuichi et al. 2015; Hohmann and Fruth 2003; great ape communication reviewed in Kalan et al. 2023). A recent report of using leaf umbrellas from the Kokolopori study site adds to this contextual bias (Samuni et al. 2022). While the different propensities in tool use appear to be independent of environmental conditions and/or social opportunities (Furuichi et al. 2015), Koops et al. (2015) found differences between chimpanzees and bonobos in the intrinsic motivation for object manipulation, a putative predisposition for the emergence of tool use. In addition, the recent work of PanAf on 144 communities of chimpanzees has shown that behavioural diversity, including tool use, is greater in environments further away from historical Pleistocene forest refugia and with more pronounced seasonality and landscape variability such as savannah locations (Kalan et al. 2020). The range of bonobos, however, is limited to the lowland forests of the Democratic Republic of the Congo (DRC) and is surrounded by geographic barriers such as water streams (IUCN 2016) which would prevent population expansion out of the forest.

Here, we report on a form of tool use from a bonobo at LuiKotale that serves water intake. Tool-aided drinking of various forms occur in wild chimpanzees, for example, at Gombe (Goodall 1964), Mahale (Matsusaka et al. 2006), Bossou (Sugiyama 1995; Tonooka 2001), Goualougo Triangle (Sanz and Morgan 2007), Ngogo (Watts 2008) and Budongo (Hobaiter et al. 2014). The work of PanAf included data on water acquisition from over 100 sites and involved forms such as moss sponges, leaf sponges and extracting water with sticks (Kalan et al. 2020), whilst Tonooka (2001) reported on leaf-folding. In addition, many chimpanzee populations have been observed to extract liquid honey using sticks (for example, Boesch et al. 2009; Kalan et al. 2020; Sanz and Morgan 2009), whilst bonobos tend to scoop it out with their fingers and hands (*personal observations*). While captive bonobos also use tools for drinking (Jordan 1982; Manrique and Call 2011), the only report of this from the wild so far comes from four observations of using moss sponges at Lomako (Hohmann and Fruth 2003). In that study, an adolescent and a juvenile female each performed the tool use three and one time, respectively, on four different occasions. By adding novel information on tool use, our report complements the behavioural repertoire of wild bonobos and contributes to the ongoing efforts to differentiate population-specific traits in the behaviour and ecology of this species.

## Methods

The field site of LuiKotale is situated in a remote area of the DRC (2° 45′ 36″ S, 20° 22′ 43″ E) and comprises lowland heterogeneous primary forest (Bessone et al. 2021). The climate at LuiKotale is equatorial with abundant rainfall (2010–2020: 1884 mm/m^2^, SD ± 225 mm, *N* = 11; Kreyer et al. 2021). Precipitation declines during the short dry season in February and a long dry season from May to August, but there is no month without rain. Research started in 2002 and involves two habituated communities: Bompusa West (habituated since 2007) and Bompusa East (habituated since 2014). At the time of the present report, communities consisted of 57 and 35 members, respectively, including infants. All members of the two communities are individually known, and all adult residents and most immatures have been genotyped. Researchers follow parties of both communities on a daily basis from nest to nest using focal animal sampling and event sampling on all adult and adolescent individuals (Altmann 1974).

Water intake is part of the ethogram and therefore recorded systematically. Here, we recorded it as an observation per day independent of the number of individuals engaging in the behaviour and of how many times it occurred.

The forest of LuiKotale is pervaded by streams and streamlets which empty into the Lokoro, a 20–30-m-wide river forming the northern border of both communities’ ranges. Accordingly, access to terrestrial sources of running water is easily available year-round, and bonobos cross streams and swamps regularly whilst making extensive use of aquatic habitats (Hohmann et al. 2019).

## Results

### Drinking behaviour

**Fig. 1:**
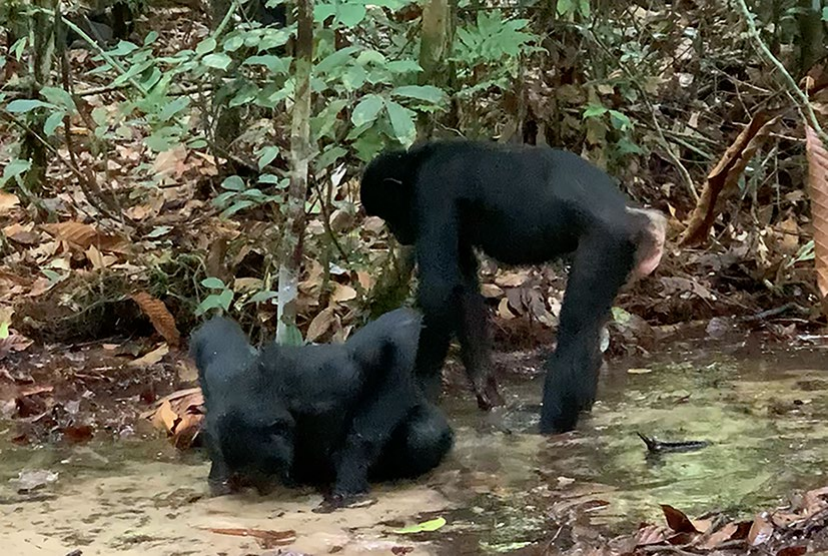
A nulliparous female drinking water from a stream in a manner typically exhibited by bonobos © LKBP/S. Pashchevskaya, 2021

**Fig. 2:**
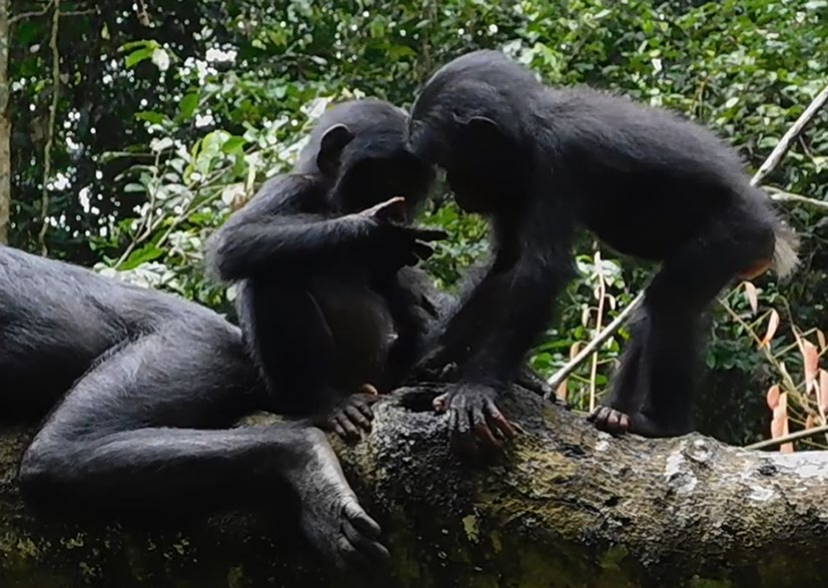
A juvenile male licking water off his hand, a juvenile female peering at him © LKBP/K. Stewart, 2019

In the year of the reported case of tool-aided water scooping, water drinking in the Bompusa West community was observed on average 5.25 times per month (range: 1–11, SD = 3.31, *N* = 63). The most common form of drinking is by lowering the head towards the surface of a stream or a pool and sucking water in (Fig. 1). Adults and immatures also tend to play with water, and in this context, individuals may suck water from the hair. In October 2019, a juvenile male bonobo was observed using his hand to scoop water out of a hole in a fallen tree in order to extract and drink water whilst a female juvenile was peering at him, after which she scooped water out of the hole herself and the male juvenile licked water off her hands (Kathrine Stewart, *personal communication*; Fig. 2). In May 2023, S.P. observed a 5-year-old female juvenile scooping water from a stream with her hand, drinking it, sucking and licking her palm.

### Tool-aided drinking

Tool-aided drinking by an adult female named Uma, a long-term resident of the Bompusa West community, was observed for the first time on 28 January 2022 by S.P. Observations started at 5:30 a.m. at the nest site. Between 7:00 and 7:30 a.m., S.P. and another field assistant separated, following two different bonobo parties. The following account was recorded by S.P.:

The initial party consisted of nine individuals: one adult male, two adult females each with an infant, one nulliparous female and three juvenile females. The party moved northeast at 7:10, and between 8:00 and 11:30 moved along the edge of a savannah, feeding on Engenkoso (*Macaranga monandra*) and unidentified *Cyperacae* species. Throughout the next few hours, there were a few changes in the party composition, and by 13:30 the party consisted of ten individuals: one adult male, two adult females with infants, one nulliparous female, one adolescent male and three juvenile females.

**Fig. 3:**
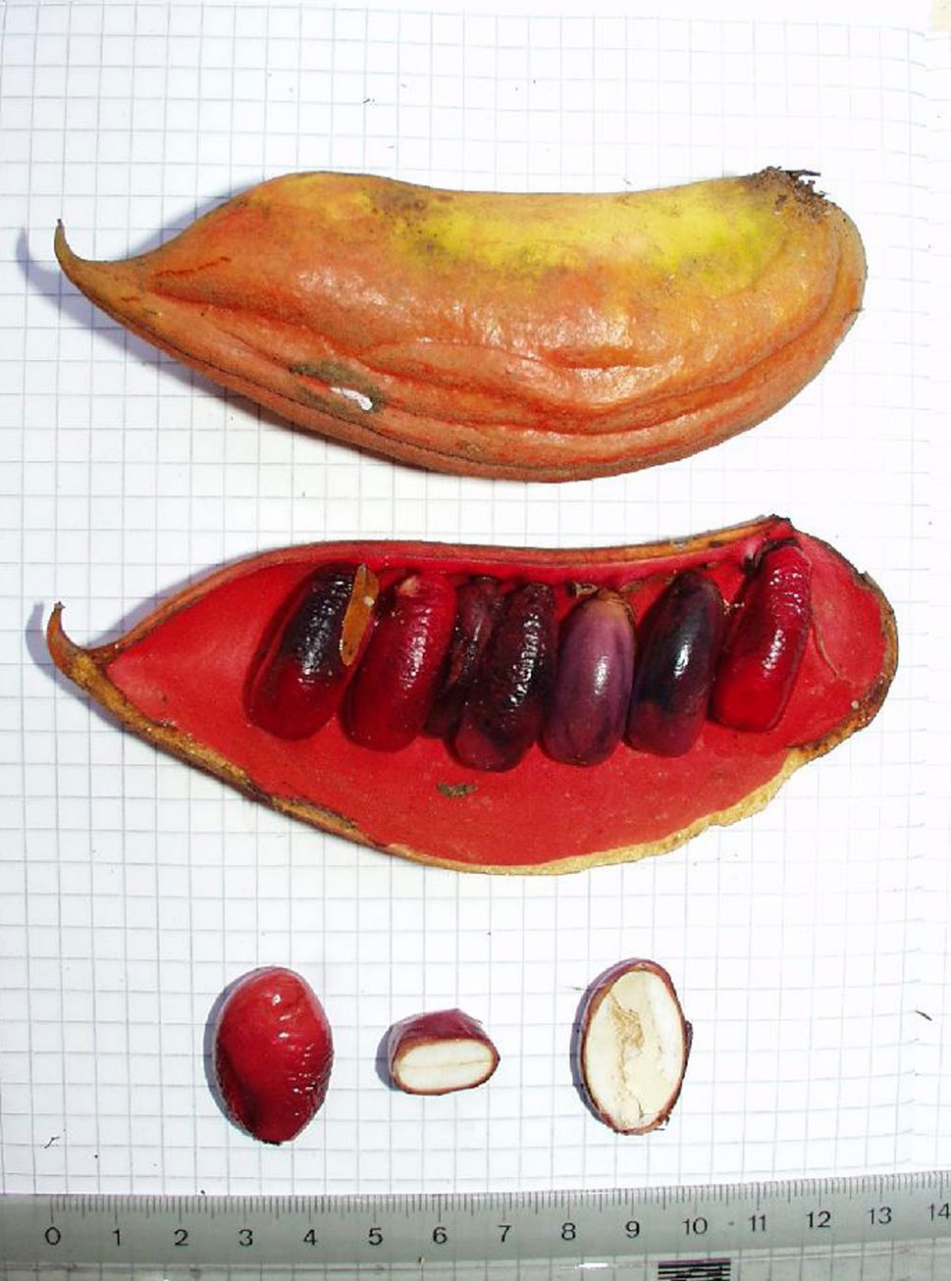
Ripe *Cola chlamydantha* pod and seeds © LKBP/B. Fruth, 2017

At 13:45, S.P. began a focal-follow of Uma as the party started moving northwest to a swampy area where they typically tend to forage for roots and mushrooms, scratching and digging the ground with their hands. In such areas, it is frequent to find small shallow streams of running water, which was also the case around 13:55. At that time, Uma obtained a cluster of ripe *Cola chlamydantha* pods (Malvaceae family, previously Sterculiaceae, Fig. 3) and proceeded to consume the seeds within. In 2022, consumption of *Cola chlamydantha* (recorded as an observation per day independent of the number of individuals consuming the food item and of how many times it occurred) was observed on average 3.42 times per month (range: 1–7, SD = 1.73, *N* = 41).

When the party stopped to rest by the stream (GPS coordinates: 2° 48.497′ S, 20° 20.265′ E, elevation 368 m), some individuals drank water by lowering their mouths to the water surface and immatures played in the water. At 14:05, Uma stopped eating *Cola* seeds and discarded the pod which, opened longitudinally, landed in the stream and filled up with water.

**Fig. 4:**
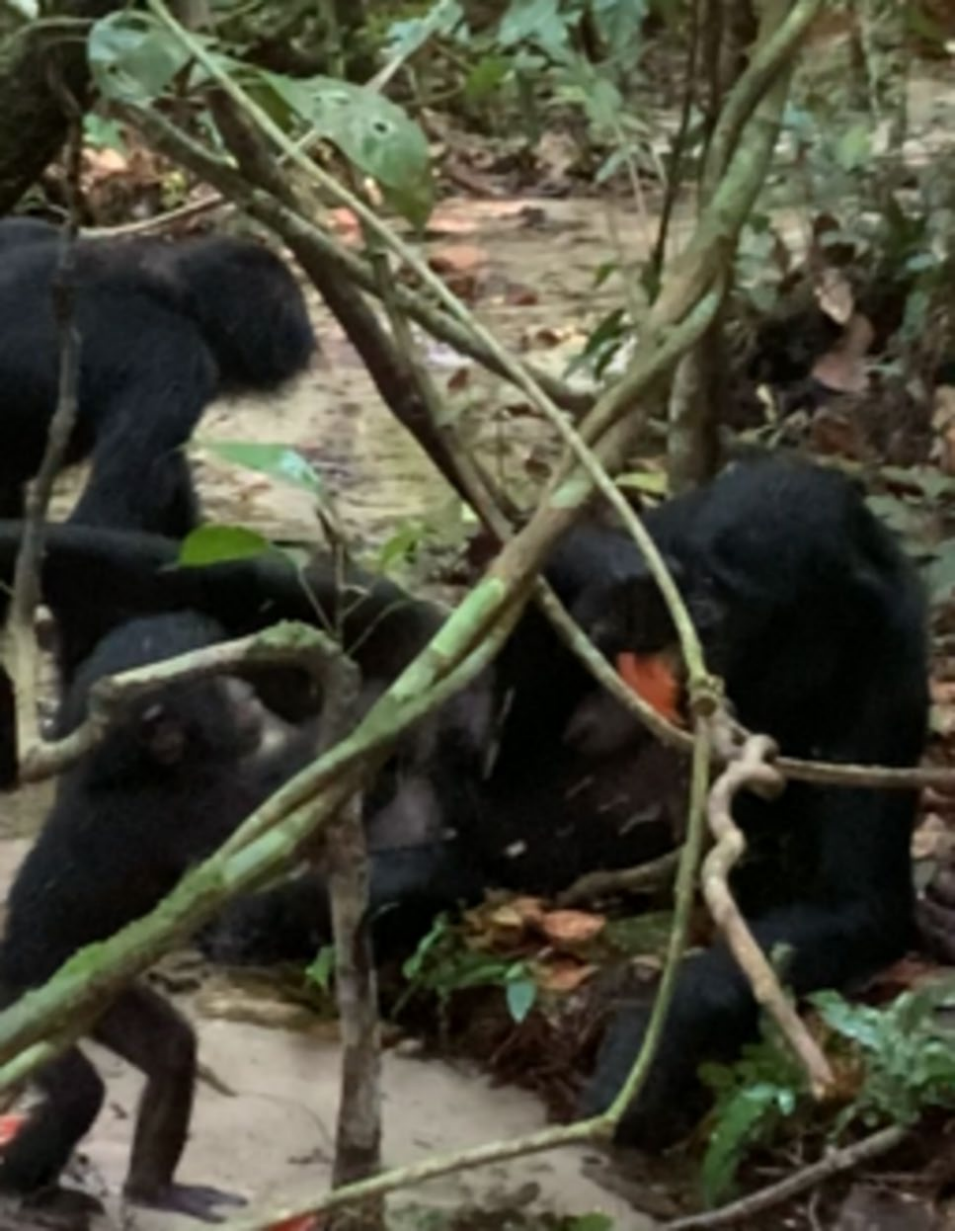
Adult female bonobo drinking water she scooped with an empty *Cola chlamydantha* pod © LKBP/ S. Pashchevskaya, 2022

Uma was lying on the ground on her left side, leaning her elbow just by the stream’s edge, her female infant Ubalda playing with Uma’s hand whilst standing in the stream. The empty open pod was between them, within Uma’s reach. After 2 min, at 14:07, Uma carefully grabbed the pod which was full of water and moved the “scoop” to her mouth (Fig. 4). Some water was being spilled, but a mouthful remained in the pod, and she drank the water from it and discarded it again, wiping her mouth with her hand (Supplementary Video 1).

At 14:10, the party started moving northwest and digging up roots and mushrooms, soon leaving the swampy area. They mostly fed on *Dialium* fruits, and around 16:30 were re-joined by one of the adult females who had left (with her infant and a male juvenile), and nested at 17:50.

## Discussion

We present a novel observation of tool-aided water intake by a wild bonobo: scooping water using discarded remains of a food item. Whilst the only other type of bonobo extractive foraging tool use observed in the wild is drinking with a moss sponge (Hohmann and Fruth 2003), “scooping” has been reported by Jordan (1982) from captive bonobos. In this case, bonobos used the pod of red pepper from their food provisions to drink water. In our case, the plant part used as a scoop has a similar shape to that of a red pepper half, but is not consumed by bonobos.

This single observation cannot yet be included in a general repertoire of tool use in wild bonobos but offers an incentive for bonobo researchers at LuiKotale and possibly other field sites to look out for similar occurrences. The main obstacle to observing this type of tool use is the low likelihood of all the relevant conditions to coincide: in our report, bonobos stopped by a stream, Uma obtained the pods, her last discarded pod landed in the water whilst she remained resting by the stream. Perhaps the majority of such inventions will never be observed by human researchers, unless it spreads to other group members and becomes part of the behavioural repertoire. Still, records on rare behaviours are informative and, in primatology in particular, have been an important source of knowledge and a prompt for new investigations (Ramsay and Teichroeb 2019; but see Sarringhaus et al. 2005 on citation errors). Such reports shed light on the innovative potential of single individuals and, eventually, the conditions that may enhance the propagation of a given behaviour. Two field assistants who engage regularly in bonobo follows collecting ad libitum data reported to us that they had seen the tool-aided water scooping, notably, in one case, performed by the same female and her sub-adult son, and in the other case, by an individual unknown by the assistant. These reports require confirmation, as it cannot be ruled out that the individuals drank the sweet viscous liquid that *Cola chlamydantha* seeds are embedded in, which is highly appreciated by humans and bonobos (Latham and Konda ku Mbuta 2014).

It is interesting to note that Uma used a tool when it was not essential to obtain the resource. The “unnecessary behaviour” of tool use for water intake was also described as habitual in immature chimpanzees in a study at Mahale, where the young chimpanzees utilised tools by the streams where they could have drunk in the “conventional” manner (Matsusaka et al. 2006). In our footage, Uma appears to be relaxed and not particularly interested in drinking, nor does she appear to purposefully manipulate the object prior to using it, but when she sees the opportunity, she acts upon it. On the other hand, as Matsusaka et al. (2006) point out, this type of drinking whilst remaining upright could offer advantages such as vigilance against predators (which did not seem to be the case in the authors’ study).

It is therefore curious to take the perspective of those debating the explanatory value of the opportunity hypothesis versus necessity hypothesis in examining innovation (Koops et al. 2014, but see Grund et al. 2019 on how the two hypotheses can be combined to explain tool use in chimpanzee). The opportunity hypothesis explains the emergence of tool use by frequent exposure to appropriate conditions and materials, whilst the necessity hypothesis proposes that tool use emerges when food is scarce and animals are motivated to obtain it from difficult-to-access sources (Fox et al. 1999), for example from tree holes (Lapuente et al. 2017; Matsusaka et al. 2006; Sugiyama 1995; Tonooka 2001). The event described in this report appears to illustrate the “opportunity hypothesis”, even if no repeated exposure to the listed conditions has been confirmed so far.

Bonobos at LuiKotale have plenty of opportunities to interact with water (Hohmann et al. 2019), in contrast to populations in more open and less humid environments (Serckx et al. 2015; Thompson 1997). Wild great apes in rainforests rarely drink water as most of it comes from food (Wrangham 1977; Rothman et al. 2008; Ashbury et al. 2015), but they depend on water more in drier and hotter savannah-like habitats as well as during increased temperatures (Wessling et al. 2018; Wright et al. 2022). Chimpanzees have been documented to dig wells to access fresh water in savannah–woodlands and savannahs (Galat et al. 2008; Hunt and McGrew 2002; Mcgrew et al. 2003; Nishida et al. 2010) and even in a rainforest (Péter et al. 2022). Whereas humans have evolved more efficient physiological water conservation (Pontzer et al. 2021), some populations of great apes may be facing challenges with respect to water intake amidst the climate change era (Wright et al. 2022; Zhang et al. 2019). In this light, the “necessity” may still arise, and it is possible that bonobos in the drier parts of the Congo Basin will be able to obtain the required resources via innovation.

In conclusion, our report illustrates the possibility of novel observations even at long-term field study sites and highlights the importance of observers’ vigilance, as such events offer grounds to speculate about species’ behavioural potential and contribute to examining population differences. Large-scale collection of new data (e.g. https://bondiv.org/) on bonobo behaviour will not only shed light on the expected intraspecific variation but also complement the rich chimpanzee behavioural diversity (Kalan et al. 2020) driven by environmental conditions.

### Supplementary Information

Below is the link to the electronic supplementary material.

Supplementary file1 (MP4 25312 KB)

## Data Availability

Non-applicable.
